# Evolving outcomes in the ICU: family ward rounds improve satisfaction year on year

**DOI:** 10.1186/cc14657

**Published:** 2015-03-16

**Authors:** R Handslip, A Molokhia

**Affiliations:** 1 University Hospital Lewisham, London, UK

## Introduction

Patient satisfaction is a crucial part of clinical care and there is now increasing recognition of the importance of family involvement and satisfaction in the provision of care for the critically ill. Since 2012 our unit has introduced a consultant-led family ward round (FWR), to enhance and standardise communication and improve satisfaction. Following Introduction of the FWR we have audited family satisfaction using the validated FS-ICU questionnaire [1].

## Methods

This was a prospective study of relatives' satisfaction for patients completing their critical care episode. The questionnaire was completed anonymously and data collected. This was a pragmatic study, no changes were made to communication strategies.

## Results

**Figure 1:**
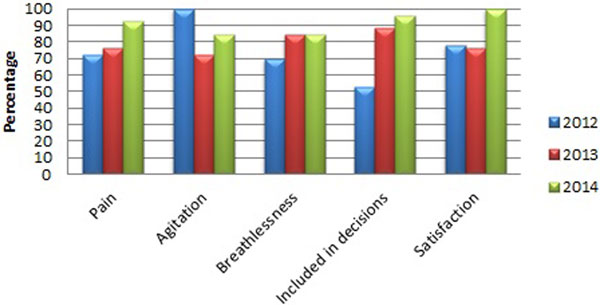
**Trends in family satisfaction**.

There is a high degree of satisfaction across all domains of the FS-ICU including treatment of family and provision of information (Figure 1). One hundred per cent found FWR to be helpful, only 55% had anticipated this. Fifteen per cent changed their perception of critical care. It enabled 15% to raise new concerns. One hundred per cent were able have questions answered satisfactorily. Linked to the FS-ICU, we have seen marked improvements in decision-making and satisfaction.

## Conclusion

We have shown progressive improvement over 3 years across all domains. Marked improvement in information provision and decision-making support from 53% to 96% over 3 years since introducing the FWR correlates with the improved overall satisfaction (Figure 1). Interestingly FWR is more helpful than relatives anticipated. The FWR was very well received and our results suggest an unrecognised need is being met. Because this was a pragmatic study, we feel this is a true representation of family satisfaction. It is encouraging that communication, information and decision-making support continue to improve. They have become embedded in the fabric of our critical care practice and lead to marked improvement in satisfaction for families.

## References

[B1] Wall Refinement, scoring, and validation of the Family Satisfaction in the Intensive Care Unit (FS-ICU) survey Crit Care Med 2007 35 271 9 17133189 10.1097/01.CCM.0000251122.15053.50

